# Prenatal diagnosis of a trisomy 7 mosaic case: CMA, CNV-seq, karyotyping, interphase FISH, and MS-MLPA, which technique to choose?

**DOI:** 10.1186/s12884-024-06522-y

**Published:** 2024-05-03

**Authors:** Xiaoyi Cong, Tong Zhang, Zhenming Li, Xiaojin Luo, Liang Hu, Weiqiang Liu

**Affiliations:** 1 Longgang District Maternity & Child Healthcare Hospital of Shenzhen City (Longgang Maternity and Child Institute of Shantou University Medical College), Shenzhen, 518172 China; 2 Longgang District Key Laboratory for Birth Defects Prevention, Shenzhen, 518172 China

**Keywords:** Low-level mosaicism, Chromosome microarray analysis, Fluorescence in situ hybridization, Karyotyping, CNV-seq, MS-MLPA

## Abstract

**Objective:**

This study aims to perform a prenatal genetic diagnosis of a high-risk fetus with trisomy 7 identified by noninvasive prenatal testing (NIPT) and to evaluate the efficacy of different genetic testing techniques for prenatal diagnosis of trisomy mosaicism.

**Methods:**

For prenatal diagnosis of a pregnant woman with a high risk of trisomy 7 suggested by NIPT, karyotyping and chromosomal microarray analysis (CMA) were performed on an amniotic fluid sample. Low-depth whole-genome copy number variation sequencing (CNV-seq) and fluorescence in situ hybridization (FISH) were used to clarify the results further. In addition, methylation-specific multiplex ligation-dependent probe amplification (MS-MLPA) was performed to analyze the possibility of uniparental disomy(UPD).

**Results:**

Amniotic fluid karyotype analysis revealed a 46, XX result. Approximately 20% mosaic trisomy 7 was detected according to the CMA result. About 16% and 4% of mosaicism was detected by CNV-seq and FISH, respectively. MS-MLPA showed no methylation abnormalities. The fetal ultrasound did not show any detectable abnormalities except for mild intrauterine growth retardation seen at 39 weeks of gestation. After receiving genetic counseling, the expectant mother decided to continue the pregnancy, and follow-up within three months of delivery was normal.

**Conclusion:**

In high-risk NIPT diagnosis, a combination of cytogenetic and molecular genetic techniques proves fruitful in detecting low-level mosaicism. Furthermore, the exclusion of UPD on chromosome 7 remains crucial when NIPT indicates a positive prenatal diagnosis of trisomy 7.

## Introduction

With the increasing use of noninvasive prenatal testing (NIPT) technology in clinical settings, screening for trisomy of chromosomes 21, 18, and 13 is now achieved with high sensitivity and specificity. This low-depth whole-genome sequencing approach has led to detection of an increasing number of rare autosomal trisomies (RATs) in the general obstetric population, with the frequency of RATs in clinical practice rangeing from 0.18–0.32% [1–3].

Trisomy 7 was the most commonly observed RAT detected by chorionic villus sampling (CVS) [4, 5]. In China, NIPT is generally performed after 12 weeks of gestation, and most prenatal diagnostic cases associated with NIPT-detected high risk for trisomy 7 refer to varying degrees of confined placental mosaicism(CPM) or true fetal mosaic status [4]. Complete trisomy 7 is lethal and can lead to spontaneous abortion before 11 weeks of gestation [6]. Therefore the fetus with trisomy 7 is usually diagnosed as mosaic [5, 7, 8].

Trisomy self-rescue is the primary mechanism to explain the high risk of RATs being prenatally diagnosed with karyotype diploidy, and it can lead to uniparental diploidy (UPD). Maternal UPD7 is related to Silver-Russell Syndrome (SRS) [9], and the UPD website has reported several UPD7 cases with or without phenotype (http://cs-tl.de/DB/CA/UPD/0-Start.html). Therefore, the impact of UPD on chromosomes 7, as well as chromosomes 6, 11, 14, 15, and 20 should be considered significant because the UPD of these chromosomes is associated with imprinted disorders [10].

Karyotyping, chromosomal microarray analysis (CMA) or low-depth whole genome copy number variation sequencing (CNV-seq), and fluorescence in situ hybridization (FISH) are the most sensitive methods for diagnosing mosaic trisomy. However, underdiagnosis of some cases with low mosaic levels has occurred when relying on the karyotyping technique [11], and a discrepancy between uncultured and cultured amniocytes has been reported in the literature [12]. Therefore, more and more centers are opting to combine karyotyping and CMA or CNV-seq for prenatal diagnosis of high-risk NIPT patients [13].

As karyotyping and CMA/CNV-seq are cytogenetic and molecular diagnostic approaches using cultured and uncultured cells, discrepancies between these methods may occur, leading to problems for healthcare providers and expectant mothers. In this study, we present a case of NIPT suggestive of trisomy of chromosome 7 with initially discordant karyotype and CMA results. Further investigation revealed a true low-level mosaic of trisomy 7, confirmed by CNV-seq and FISH analysis, and the majority of cells were excluded from UPD7 as indicated by methylation-specific multiplex ligation-dependent probe amplification (MS-MLPA).

## Materials and methods

### Study subjects

A 28-year-old woman, gravida 2 and parity 0, was found to be at high risk for Down syndrome after serum screening and underwent NIPT at 13^+ 1^ weeks. She had no family history of congenital malformations, drug or radiation exposure, abdominal pain, or vaginal bleeding. Amniocentesis was performed at our medical center at 17^+ 1^ weeks. Subsequently, the karyotyping and CMA were performed on the amniotic fluid sample. Due to the inconsistent results of the two methods, amniotic fluid samples were subjected to CNV-seq, FISH, and MS-MLPA analysis during repeat amniocentesis. This study was approved by the Medical Ethics Committee of Shenzhen Longgang District Maternal and Child Health Hospital(No. LGFYYXLLQF-2022-003), and informed consent was obtained from the participants before all tests.

### Karyotyping analysis

Amniotic fluid cells were aseptically inoculated into two independent cell culture flasks and cultured in a 37 °C, 5% CO2 incubator for 6–8 days, depending on their growth status. Chromosome preparation was performed by conventional methods. Karyotypes were scanned using an automated Leica machine, and only well-distributed banded clones with chromosomes having no or relatively few (≤ 5) crossovers were selected for analysis.

### CMA

DNA was extracted using the QIAamp DNA Blood Mini-Kit (No. 51106, Qiagen, Germany). The Affymetrix Cytoscan 750 K chip (Thermo Fisher Company) was used. According to the instructions, the extracted DNA was digested, ligated, PCR amplified, purified, fragmented, and labeled for hybridization. After washing and staining, the chip was scanned, and the data were collected and analyzed using ChAS4.3 software. The copy number variants(CNVs) and region of homozygous(ROH) status were analyzed by setting the threshold of 100 Kb for CNV and 5 Mb for ROH.

### CNV-seq

DNA libraries were prepared by enzymatic cleavage of DNA strands, DNA end repair, ligation of junctions, and amplification by PCR. An average sequencing depth of 0.1× was required using the GeneMind GenoLab M sequencing platform (GeneMind Company, China). Sequencing results were then compared to the human reference genome (hg19). Chromosomal variants were analyzed by querying databases such as DECIPHER and OMIM.

### FISH

FISH was performed on 5 ml of uncultured amniotic fluid. A prenatal chromosome detection kit from Abbott was used for hybridization, washing, re-staining, and placing the slides under the fluorescence microscope according to the instructions in the manual. More than 100 cells were counted.

### MS-MLPA

MS-MLPA analysis was performed using the SALSA MLPA reagent kit (ME034, MRC-Holland, The Netherlands) according to the manual, and the PCR product was then loaded onto a 3500Dx generation sequencer (Thermo Fisher Company, USA) for capillary electrophoresis analysis.

## Results

### Karyotyping

**Fig. 1:**
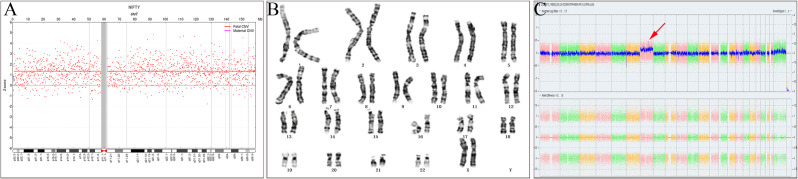
High-risk of trisomy 7 indicated by NIPT and the result of invasive prenatal diagnosis (**A**) NIPT suggests the presence of trisomy 7, with a Z score of 28.037; (**B**) karyotyping of amniotic fluid cells (more than 100 schizogony phases) showed normal diploidy results; (**C**) genome-wide chromosomal microarray analysis detected a mosaic trisomy 7, and SNP signal did not find any ROH

As the NIPT result was positive for trisomy 7 (Fig. 1A), microscopic analysis of 20 schizogony phases of cultured amniotic fluid cells revealed a karyotype of 46, XX. Further analysis of 100 additional schizogony phases confirmed this result (Fig. 1B).

### CMA

The CMA test showed a mosaic trisomy 7, with increased copies observed for this chromosome. The ChAS software showed a smooth signal value of 2.2 for chromosome 7, indicating that approximately 20% of the cells were trisomy 7 and 80% were diploid(Fig. 1C).

### CNV-seq

**Fig. 2:**
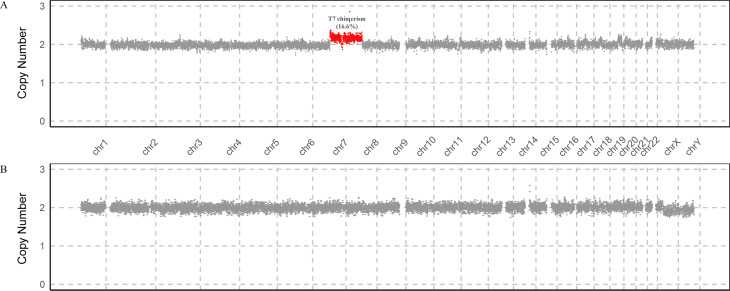
CNV-seq results (**A**) CNV-seq identified a 16% mosaic trisomy 7; (**B**) Diploidy results from a healthy control sample

The pregnant woman underwent repeat sampling two weeks later due to discordant karyotype and CMA results, and the CNV-seq result of uncultured amniotic fluid cells suggested mosaic trisomy 7 with a frequency of approximately 16% (Fig. 2).

### FISH results

**Fig. 3:**
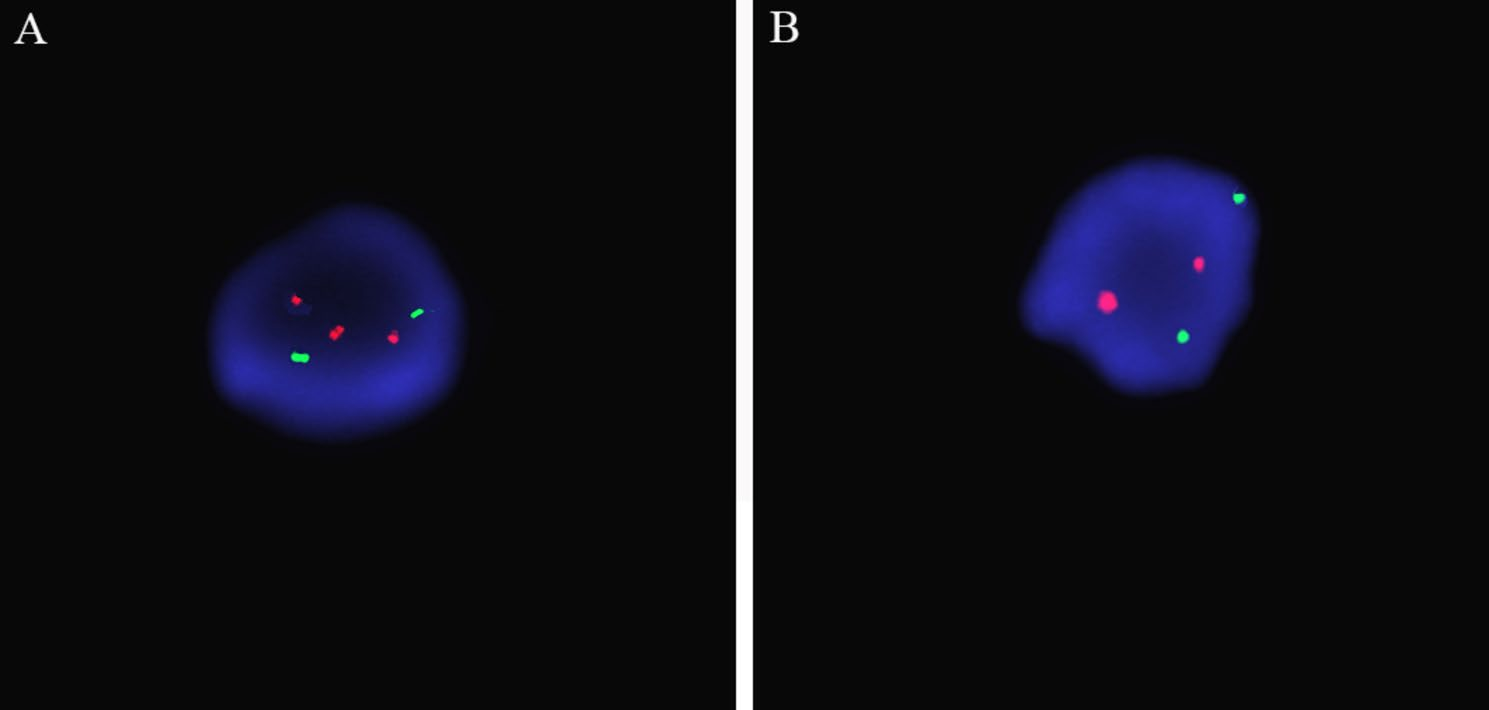
FISH showing a low-level mosaic trisomy 7 (**A**) The mitotic probe showed three red signals in the cell, representing three copies of chromosome 7, the trisomy signal in 4% of the total 100 cells analyzed; (**B**) two red signals represent a normal diploid cell. The green signals indicate a standard diploid control of chromosome 13

To further confirm or exclude mosaicism, FISH showed that out of 100 uncultured interphase cells, four cells had three specific signals on chromosome 7, indicating that 4% of the cells were trisomy 7 ( Fig. 3).

### MS-MLPA results

**Fig. 4:**
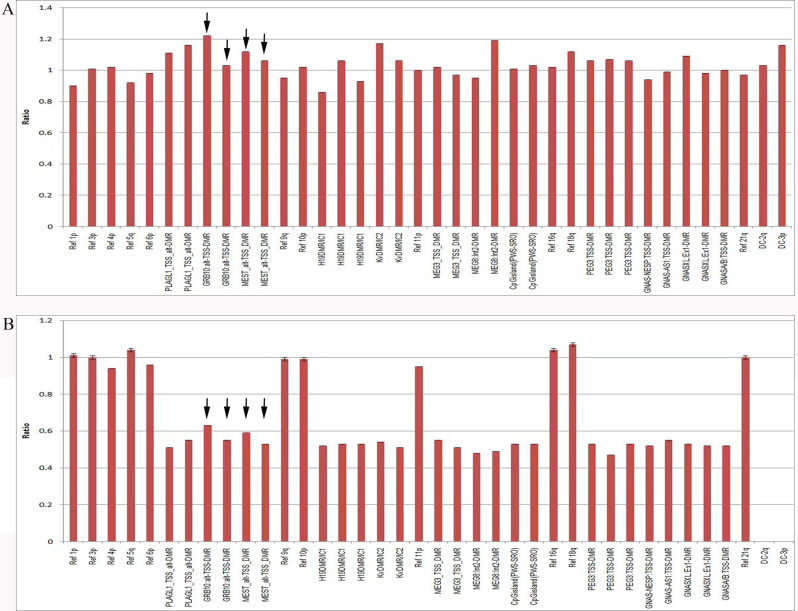
MS-MLPA of the mosaic trisomy fetus (**A**) The results of the MS-MLPA show no detectable copy number changes in the imprinted region covered by the chromosome 7 probe, although there is only one proble of GRB10 has a slight copy number signal higher than threshold 1.2; it has no significant increase compared to the other probes; (**B**) The results of the MS-MLPA show no detectable methylation abnormality in the imprinted region covered by all four probes on the chromosome 7

The MS-MLPA results show no detectable copy number changes, as the final ratio of most probes on chromosome 7 is between 0.8 and 1.2, indicating a diploid status. Although one probe of GRB10 has a slight copy number signal higher than 1.2, it has no significant increase compared to the other probes (Fig. 4A). In addition, the signal of the methylation status signal of the four probes (two probes for GRB10 at 7q12.2 and two probes for MEST at 7q32.2) were all within the normal range (Fig. 4B), suggesting that no methylation abnormalities were observed in this case.

### Ultrasound observation

No significant abnormalities in fetal growth, development, or congenital malformations were observed at the subsequent ultrasound examinations. However, at 39 weeks of gestation, the ultrasound revealed that the fetus had developed a mild condition of growth restriction. The ultrasound observation showed the biparietal diameter(BPD) was 8.60 cm(8.69–9.89 cm), head circumference(HC) was 31.5 cm(32.25–36.25 cm), femur length(FL) was 6.90(6.97–8.17 cm), abdominal circumference(AC) was 31.80 cm(31.63–36.99 cm) when the fetus was 39th weeks of gestation.

### Pregnancy and postpartum outcome

After genetic counseling, the pregnant woman decided to continue the pregnancy. The fetus was born at 39^+ 5^ weeks gestation, and weighed 2.35 kg. After birth, the skin color was reddish, the spontaneous breathing, crying, and muscle tension were normal, and the Apgar score was 10 at 1 min and 5 min, respectively. The second follow-up was performed when the baby was three months old, and the infant’s growth and development were normal.

## Discussion

Trisomy 7 is one of the most common aneuploidies detected by CVS [4, 5]. Complete trisomy of chromosome 7 is typically considered a lethal embryonic abnormality [14]. Clinical live births with trisomy 7 are rare and almost always detected as mosaics [15]. The postnatal individuals with mosaic trisomy 7 presented with diverse clinical phenotypes, ranging from unremarkable to symptoms such as radial defects, pulmonary dysplasia, hypomelanosis of Ito, facial dysmorphism, enamel dysplasia, pigmentary abnormalities, Potter syndrome, Goldenhar syndrome, and Blaschkolinear malformation syndrome [16–18].

The use of karyotyping or rapid molecular diagnosis (QF-PCR) in prenatal diagnosis can clarify most high-risk cases of trisomy 21, 13, and 18 indicated by NIPT, and the positive predictive value (PPV) of screening for trisomies 21, 18, and 13 is as high as 80% or more [19]. However, the PPV of NIPT indicates a risk of RATs of only 4.1–6%, according to two extensive studies [2, 3], which is mainly due to the self-rescue mechanism of trisomies in early embryonic stages, resulting in an aneuploid fetus and a trisomic placenta. Incomplete trisomic self-rescue processes may result in the presence of uniparental disomy or uniparental disomy with a low level of mosaic trisomy in the fetus [20].

The detection rate for cases of mosaic trisomy 7 has increased with the widespread use of NIPT [5, 21]. When chromosomal karyotyping alone was used, positive diagnoses could be missed [22]. Prenatal diagnosis for high-risk NIPT is mainly based on karyotype combined with CMA or CNV-seq techniques. However, the combined use of cytogenetic and molecular genetics in clinical practice is challenged by inconsistent results between these methods [23–25], leading to significant confusion in clinical diagnosis and genetic counseling. One reason for discrepancies in results between cultured and uncultured amniocytes is the potential loss or incompleteness of cultured cells during culture flask and titration harvest. Although molecular diagnostic techniques such as CMA and CNV-seq use simple cells for direct DNA extraction, this can also lead to inconsistencies between karyotype and CMA or CNV-seq results due to method sensitivity or mosaic detection limits.

In this study, the pregnant woman underwent prenatal diagnosis after NIPT, which indicated a high risk of trisomy 7. The amniotic fluid karyotype results were normal diploidy after careful analysis of 100 schizogony phases. The CMA showed a 20% level of mosaic trisomy 7, in contrast to the karyotyping results. The patient was re-punctured, and CNV-seq and FISH analyses were performed. CNV-seq detected a 16% mosaic level of trisomy 7, consistent with the CMA results. However, FISH detected a mosaic rate of only 4%, lower than CMA and CNV-seq. This difference can be explained by the fact that CMA and CNV-seq use a more cells for DNA extraction, while FISH detects individual cells one at a time. Nevertheless, all three methods revealed the presence of mosaic trisomy 7 in amniotic fluid samples, indicating that the fetus is indeed mosaic trisomy 7.

Trisomy self-rescue can result in UPD on chromosome 7 because it contains an imprinted region. Maternally derived UPD (7) is associated with SRS, which is primarily characterized by prenatal and postnatal growth restriction, macrocephaly, prominent forehead, triangular phases, small jaws, and dental anomalies [26, 27]. Approximately 7–10% of individuals with SRS have UPD of maternal origin on chromosome 7, whereas UPD (7) of paternal origin typically does not affect growth or development [28]. Therefore, it is essential to monitor for low levels of mosaic trisomy and also perform methylation detection to confirm or exclude the presence of UPD when NIPT indicates a high risk of aneuploidy on chromosomes 6, 7, 11, 14, 15, or 20, as imprinted disorders may occur in the presence of UPD [29].

In our low-level mosaic trisomy 7 case, most of the cells are diploid, accounting for more than 80% of the total cells, while trisomic cells accounted for less than 20%. As mentioned above, self-repair of trisomy 7 may result in UPD or diploidy/trisomy mixed cells; therefore, in this study, we used the MS-MLPA method to check whether UPD status is present in this case. Interestingly, the MS-MLPA results did not show any significant copy number changes, although only one probe of GRB10 showed a slightly higher signal, and no methylation abnormalities were observed in this case, as the signal of all four probes was within the normal range. Although MS-MLPA is sensitive for methylation analysis, our data revealed demonstrated the limitations of this technique when dealing with low-level trisomy.

Genetic counseling for mosaic trisomy at amniocentesis is challenging due to the condition’s phenotypic variability of the condition, with some fetuses displaying the typical phenotype and others appearing normal. Overall, the severity of the clinical phenotype is influenced by the proportion and location of mosaicism. As the proportion of abnormal cells increases, so does the severity of the phenotype. However, identifying the location of the mosaicism in the prenatal setting is challenging, and caution must be exercised when inferring the severity of the clinical phenotype from the proportion of mosaicism.

The presence of uniparental disomy 7 not detected in the NIPS trisomy 7-positive pregnancies with normal fetal karyotype has been reported [30]. In this study, the majority of cells in our case could be excluded as UPD based on the MS-MLPA data, and CMA did not observe any ROH on chromosome 7, further confirming this conclusion. Fetal follow-up was normal, further excluding the possibility of UPD 7. The slight growth delay observed at 39th weeks of gestation may be caused by the trisomy 7 on the placenta.

In conclusion, we have successfully detected a case of true low-level mosaic trisomy 7 using cytogenetic and molecular genetic diagnostic techniques. For individuals at high risk of trisomy based on NIPT, we recommend simultaneous testing with cytogenetic and molecular genetic approaches to reduce the risk of underdiagnosis of low-level mosaic cases. Different techniques such as CMA, CNV-seq, FISH, and chromosomal karyotyping have their strengths and weaknesses for testing low-level mosaic cases, and the combined use of multiple methods can accurately differentiate between true and false mosaic cases [12]. For rare autosomal trisomies associated with imprinted disorders, such as high-risk trisomies 6, 7, 11, 14, 15, and 20, additional tests such as MS-MLPA should be performed to exclude the likelihood of imprinted disorders.

## Data Availability

The data sets generated and analyzed in the current study are available in the SRA repository (https://www.ncbi.nlm.nih.gov/sra/PRJNA1058142) under the accession number: PRJNA1058142.
